# Assisted Phytostabilization of Mine-Tailings with *Prosopis laevigata* (Fabaceae) and Biochar

**DOI:** 10.3390/plants11243441

**Published:** 2022-12-09

**Authors:** Juan Ramírez-Zamora, Patricia Mussali-Galante, Alexis Rodríguez, María Luisa Castrejón-Godínez, Leticia Valencia-Cuevas, Efraín Tovar-Sánchez

**Affiliations:** 1 Doctorado en Ciencias Naturales, Universidad Autónoma del Estado de Morelos, Av. Universidad 1001, Col. Chamilpa, Cuernavaca CP 62209, Mexico; 2 Laboratorio de Investigaciones Ambientales, Centro de Investigación en Biotecnología, Universidad Autónoma del Estado de Morelos, Av. Universidad 1001, Col. Chamilpa, Cuernavaca CP 62209, Mexico; 3 Facultad de Ciencias Biológicas, Universidad Autónoma del Estado de Morelos, Av. Universidad 1001, Col. Chamilpa, Cuernavaca CP 62209, Mexico; 4 Centro de Investigación en Biodiversidad y Conservación, Universidad Autónoma del Estado de Morelos, Av. Universidad 1001, Col. Chamilpa, Cuernavaca CP 62209, Mexico

**Keywords:** biochar, phytostabilization, mine tailings, heavy metals, *Prosopis laevigata*

## Abstract

Phytoremediation is a cost-effective technique to remediate heavy metal (HM) polluted sites. However, the toxic effects of HM can limit plant establishment and development, reducing phytoremediation effectiveness. Therefore, the addition of organic amendments to mine wastes, such as biochar, improves the establishment of plants and reduces the bioavailability of toxic HM and its subsequent absorption by plants. *Prosopis laevigata* can establish naturally in mine tailings and accumulate different HM; however, these individuals show morphological and genetic damage. In this study, the effect of biochar on HM bioaccumulation in roots and aerial tissues, HM translocation, morphological characters and plant growth were evaluated, after three and six months of exposure. Plants grown on mine tailings with biochar presented significantly higher values for most of the evaluated characters, in respect to plants that grew on mine tailing substrate. Biochar addition reduced the bioaccumulation and translocation of Cu, Pb, and Cd, while it favored the translocation of essential metals such as Fe and Mn. The addition of biochar from agro-industrial residues to mine tailings improves the establishment of plants with potential to phytoextract and phytostabilize metals from polluted soils. Using biochar and heavy metal accumulating plants constitutes an assisted phytostabilization strategy with great potential for HM polluted sites such as Cd and Pb.

## 1. Introduction

Worldwide, mining is among the most important productive activities which provides strategic factors for economic development and wealth generation [1,2]. Mexico is among the main global producers of 17 different minerals and has been the first in silver production for more than 12 consecutive years. However, despite its economic and social benefits, mining is recognized as one of the most environmentally disturbing activities, due to the great amount of hazardous waste that it generates, mainly in the processes related to the extraction of valuable ores from non-economic value materials [3,4]. Mining releases to the environment diverse pollutants, including several gases, wastewater, and mine tailings [5]. These wastes contain several toxic HM, which are non-degradable and persistent in the environment, causing soil and water pollution and threatening wildlife and human health [6,7].

Mine tailings are finely ground solid waste generated because of rock trituration processes in the recovery of the economic value metals. These materials are commonly deposited without treatment as mounds on the side of mines or submerged as sludge in tailing dams [3]. Mine tailings are commonly found in the surroundings of abandoned mining districts and small-scale or artisanal mining operations. Contact of mine tailings, with rain and winds, favors pollutant release and dispersion in the surrounding areas [8]. Therefore, active or abandoned mine tailings are recognized as one of the primary sources of HM pollution [9].

The presence of metals in the environment can exert adverse effects on the ecosystems because of their bioaccumulation in different organisms. Plants can uptake HM from the soil through the root system and some metals may be translocated to the aerial tissues [10]. Then, these plants are consumed by different herbivores resulting in metal entrance and transfer along the trophic webs causing HM biomagnification [11]. Hence, the remediation of HM polluted sites has been of great relevance in the last decades [12,13,14,15].

In this context, bioremediation has been proposed as an effective alternative for metal cleaning, using different organisms such as bacteria, yeasts, fungi, algae, and plants [16,17]. The use of plants for removing pollutants from the environment is known as phytoremediation [18,19,20], which is an economical, efficient, and environmentally friendly approach [21,22].

For successful phytoremediation, plants must be capable of tolerating HM exposure by exclusion, stabilization, and by accumulating them in the aerial tissues [23,24,25]. Moreover, mine-tailing phytoremediation can be carried out in situ, thus reducing the risks of secondary contamination in the handling and transporting of polluted materials. However, there are some limitations for the application of phytoremediation approaches; the physical, chemical, and biological properties of these wastes affect the establishment and growth of plants [21,26], metal toxicity causes a reduction in size and other morphological and physiological characters [23,27], it is restricted to the surface and depth of the root [28], and phytoremediation is a slow process, that could take several years, in which favorable climatic conditions for plant development are required [20].

To overcome such phytoremediation disadvantages, several assisted phytostabilization strategies have been proposed, including the addition of different organic amendments to the polluted substrates [29,30]. These amendments modify the physicochemical properties of the substrates, improve their structure, provide nutrients, and lead to a greater water retention capacity [31,32,33]. Among the amendments used for this purpose, biochar is particularly effective [34]. Biochar is the product obtained from thermal degradation at low levels or in the absence of oxygen (pyrolysis) of different organic materials [35,36]. In addition, biochar reduces the bioavailability of HM and metalloids and their absorption by plants because they can interact with metals through various mechanisms, such as electrostatic interactions, ion exchange, sorption via π electron displaced carbon, surface precipitation or co-precipitation, and adsorption [37]. These interactions allow the biochar to retain metals in the soil, considerably reducing their concentration in plant tissues [35,36]. In addition, the alkaline pH of biochar increases the pH of the soil into which it is incorporated [34], reducing the solubility and bioavailability of metals [38].

*Prosopis laevigata* (Humb. et Bonpl. ex Willd) M.C. Johnston (Fabaceae) is an arboreal species with a broad worldwide distribution in arid and semi-arid zones. In addition, this plant can establish itself in variable temperature and moisture conditions and grow in nutrient-poor soils [39,40]. Some studies show that it settles in mine tailings with high HM and metalloid contents and is capable of accumulating Al, As, Cd, Cr, Cu, Fe, Mg, Ni, Pb, V, and Zn [23,41,42,43,44,45]. However, *P. laevigata* shows significant adverse effects due to the bioaccumulation of several HM in root and leaf tissues, such as genetic damage and reductions in several morphological and physiological characters [23]. Therefore, the aim of this study was to evaluate the effect of biochar when mixed with mine tailings to (1) evaluate the establishment of *P. laevigata* in tailing substrates (in situ establishment); (2) to evaluate if this assisted phytostabilization can reduce the accumulation and translocation of metals in plant tissues; (3) to evaluate if exposure to mine tailing and mine tailing/biochar substrates cause changes in size and biomass of *P. laevigata* individuals. This approach will allow us to determine if biochar from coconut fiber and corn cob can be an appropriate methodology to facilitate the establishment of this particular HM accumulator plant for phytostabilization of polluted soils, especially those polluted with the non-essential and most toxic HM.

## 2. Results

### 2.1. Physicochemical Characterization of the Corncobs/Coconut Fiber Biochar

According to the physicochemical characterization, the biochar (50% corn cobs/coconut fiber mixture), employed in the present research as an organic amendment, showed a pH value of 10, 4.4% of OM, 10.5 mg/kg of phosphorous, 0.033 mg/kg of nitrogen, 2.6 mg/kg of carbon, and C/N ratio of 78.8. According to the reference values established in the Mexican standard, NOM-021-SEMARNAT-2000, the corn cobs/coconut fiber biochar is a strongly alkaline substrate with a high OM proportion. In contrast, concerning the C/N ratio and phosphorous content the observed values were situated in the medium range.

### 2.2. Effect on the Mine Tailing Exposure and the Biochar Addition over the Size Characters of P. laevigata

**Figure 1:**
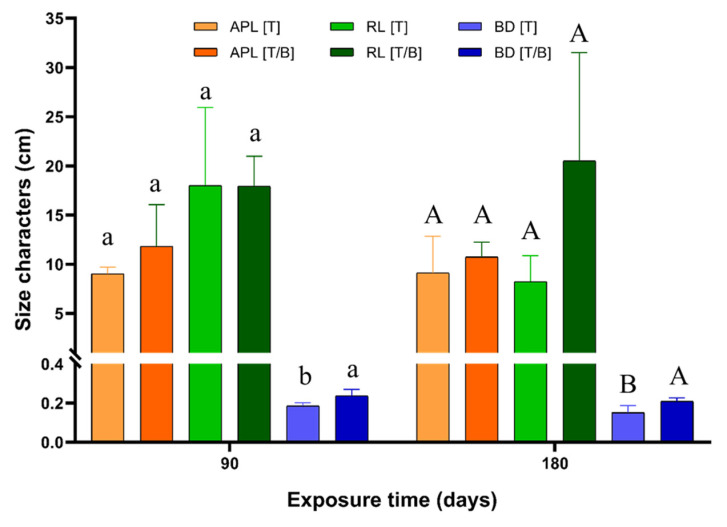

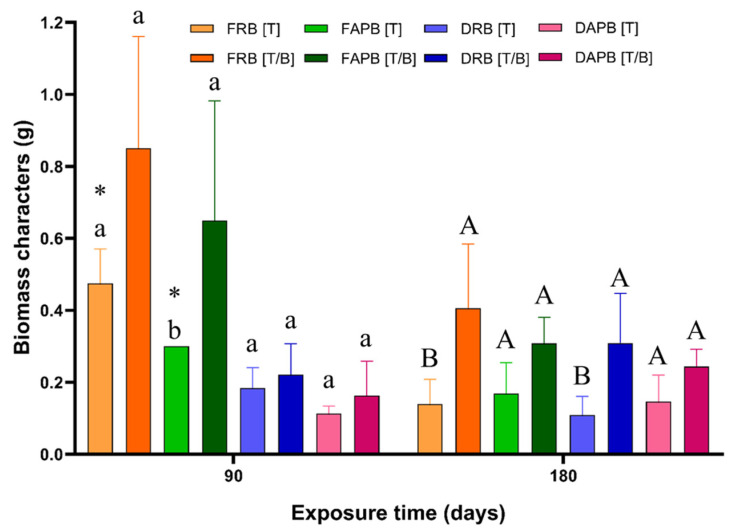
Size and biomass characters for *P. laevigata* growing on tailing (T) and tailing/biochar (T/B) in in situ conditions during 90 and 180 days. APL = aerial part length, RL = root length, BD = basal diameter, FRB = fresh root biomass, FAPB = fresh aerial part biomass, DRB = dry root biomass, DAPB = dry aerial part biomass. Different lowercase letters denote significant differences among treatments after 90 days of the exposure (Tukey *p* < 0.05). Different uppercase letters denote significant differences among treatments after 180 days of the exposure (Tukey *p* < 0.05). * = statistical differences between exposure times for each character.

After three months of exposure, *P. laevigata* plants growing on mine tailing–biochar substrate showed significantly higher values in the basal diameter and fresh biomass of the aerial tissues with respect to the plants growing on mine tailing substrate. Similar results were observed after six months of exposure, the plants that were grown on the mine tailing–biochar substrate showed higher values in the basal diameter and the fresh and dry biomass of the roots in comparison to the plants growing on the mine tailing substrate (Figure 1, Table S1). Because of the heavy metal exposure, the plants growing on mine tailing substrate showed a significant reduction in the fresh biomass of the root and the aerial tissues through exposure time, while the plants growing on mine tailing substrate supplemented with biochar did not show significant changes in their size characters over the exposure time (Figure 1, Table S1).

### 2.3. Bioaccumulation and Translocation Profiles of Nonessential Heavy Metals in P. laevigata

The bioaccumulation of two non-essential heavy metals, Cd and Pb, was evaluated in the roots and aerial tissues of *P. laevigata* plants exposed to two treatments: individuals growing on mine tailings and mine tailings supplemented with 12% of corncobs/coconut fiber biochar. Two samples were obtained to evaluate the effects of the respective treatment and the exposure time of three and six months.

After three months of exposure, no significant effect of the treatments (plants growing on mine tailings and mine tailings supplemented with biochar) over the bioaccumulation of Cd in the roots of *P. laevigata* individuals was observed. However, after six months of exposure to the mine tailing substrate, *P. laevigata* individuals bioaccumulated a higher Cd concentration in roots. The exposure time showed a significant effect on Cd bioaccumulation in the roots of plants growing on the mine tailing substrate. However, the Cd concentration in the roots of *P. laevigata* plants growing on the substrate supplemented with biochar did not increase over time.

**Figure 2:**
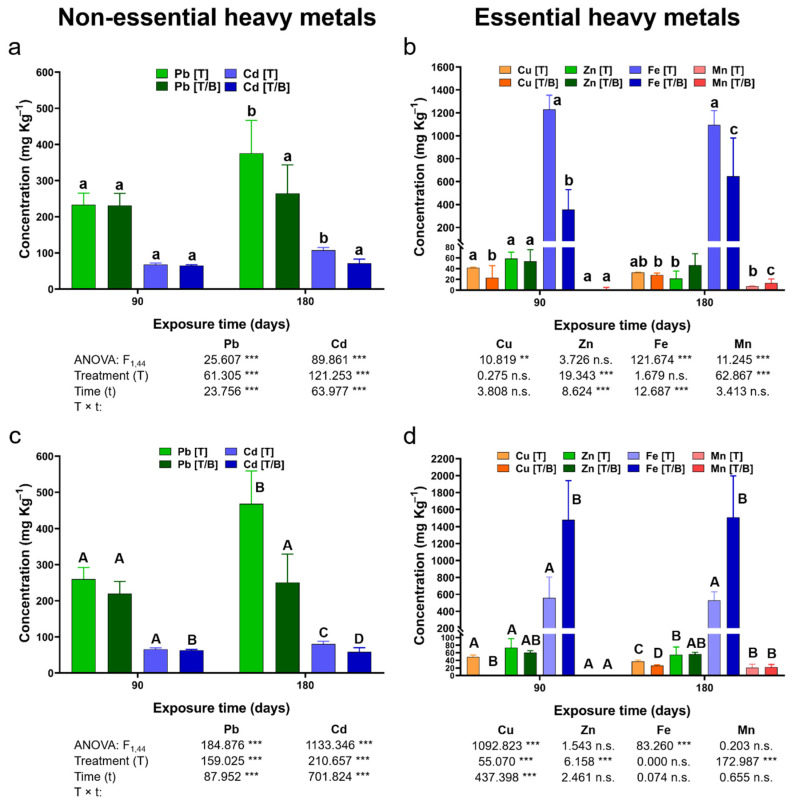
Average ± standard deviation and two-way ANOVA results for heavy metal concentration (mg Kg^−1^) in roots (**a**,**b**) and leaves (**c**,**d**) of *P. laevigata* growing on tailing (T) and tailing/biochar (T/B) in in situ conditions during 90 and 180 days. Different lowercase letters denote significant differences among treatments in heavy metal root concentration (Tukey *p* < 0.05). Different uppercase letters denote significant differences among treatments in heavy metal leaves concentration (Tukey *p* < 0.05), n.s. = not significant differences, ** = *p* < 0.01, *** = *p* < 0.001.

The plants exposed to the mine tailing substrate bioaccumulated significantly higher Cd in the aerial tissues at three and six months of exposure, compared to Cd bioaccumulation in the aerial tissue of plants exposed to the biochar supplemented mine tailing substrate. Moreover, Cd concentration in the aerial tissues of *P. laevigata* growing on the mine tailing substrate significantly increased over time. In contrast, the plants growing in the presence of the coconut/corncob biochar, showed a reduction in Cd concentration over time, in the same tissue (Figure 2, Table S2). However, Cd translocation factors (TF) were lower than one for both treatments (plants growing on mine tailings and mine tailings supplemented with biochar).

With respect to Pb bioaccumulation in the roots of *P. laevigata*, no significant effect of the treatments was observed after three months of exposure. However, Pb concentration in roots increased significantly in *P. laevigata* individuals after six months of exposure to the mine tailing substrate. The plants exposed to the mine tailing substrate showed a significant increase in Pb concentration in the roots over time. In contrast, exposure time did not show a significant effect on Pb bioaccumulation in the roots of the plants established in the mine tailing substrate with the presence of coconut fiber/corncobs biochar (Figure 2, Table S2).

Three months after the establishment of *P. laevigata* in the treatments, no significant effect was detected on the bioaccumulation of Pb in the aerial tissues of the plants. While after six months of exposure, it was documented that the plants established in the mine tailing substrate, bioaccumulated a significantly higher Pb concentration in the aerial tissues. Plants of *P. laevigata* established in the mine tailing substrate showed a significant increase in Pb concentration in the aerial part over time. In contrast, the exposure time did not significantly affect Pb bioaccumulation in the aerial tissues of the plants growing in the presence of coconut/corncobs biochar (Figure 2, Table S2). After three and six months of exposure, the TF values observed in plants growing in mine tailing substrate were higher than one, while in the plants growing in the presence of corncobs/coconut fiber biochar, the TF showed values lower than one in both sampling times, three and six months.

### 2.4. Bioaccumulation and Translocation Profiles of Essential Heavy Metals in P. laevigata

The bioaccumulation of four essential heavy metals, Cu, Fe, Mn, and Zn was evaluated in the roots and aerial tissues of *P. laevigata* plants exposed to mine tailing substrate and mine tailings supplemented with 12% of corncobs/coconut fiber biochar. Roots and leaf samples were obtained after three and six months of exposure.

After three months, the plants growing on mine tailing substrate bioaccumulated significantly higher Cu in roots than Cu concentrations in the roots of those plants growing on the presence of the corncobs/coconut fiber biochar. However, after six months, Cu bioaccumulation in the roots of plants growing on both treatments did not show significant differences. Hence, the exposure time did not affect Cu bioaccumulation in *P. laevigata* roots regardless of the treatment (Figure 2, Table S2).

Copper bioaccumulation in aerial tissues of *P. laevigata* was significantly higher in the plants exposed to the mine tailing substrate at two sampling times, three and six months, when compared to Cu bioaccumulation in the aerial tissues of the plants growing on the presence of corncobs/coconut fiber biochar. However, Cu bioaccumulation in the aerial tissue of the exposed plants to mine tailing, decreased from three to six months, while in the plants exposed to biochar supplemented with mine tailing substrate, Cu bioaccumulation increased over time. Finally, the TF determined at both sampling times in the plants growing on mine tailing substrate showed values higher than one, indicating the translocation of Cu from roots to the aerial tissues, while in the plants growing on biochar supplemented with mine tailing substrate, Cu translocation was reduced, resulting in TF values lower than one (Figure 2, Table S2).

Iron bioaccumulation in the aerial tissues of the plants exposed to mine tailing substrate was significantly higher in comparison to Fe bioaccumulation in the aerial tissues of plants growing on the mine tailing substrate with biochar at two sampling times. Iron bioaccumulation in aerial tissues did not increase over time in the plants growing on both treatments. With respect to Fe translocation, in the plants exposed to mine tailing substrate, TF values were higher than one at the two sampling times, while in the plants growing in the presence of corncobs/coconut fiber biochar, TF values were lower than one (Figure 2, Table S2).

After three months of exposure, the bioaccumulation of Mn in the roots of *P. laevigata* plants was not affected by the treatment, while after six months of exposure, the plants growing on the presence of biochar bioaccumulated higher Mn concentrations in the roots. However, in both treatments, the plants showed significant increases in Mn concentration in the roots over time (Figure 2, Table S2).

Concerning Mn bioaccumulation in the aerial tissues of *P. laevigata*, the plants growing on both treatments increased Mn concentrations over time. However, Mn concentrations in the aerial tissues were similar in the plants of both treatments after three and six months of exposure. TF values in the plants exposed to mine tailing substrate were higher than one at the two sampling times, while in the treatment with biochar, after three months of exposure the plants showed TF values lower than one, but after six months of exposure, the TF increased to greater than one (Figure 2, Table S2).

The plants growing on mine tailing substrate and those growing on the substrate with biochar bioaccumulated similar Zn concentrations in the roots after three months of exposure. However, after six months of exposure, *P. laevigata* individuals growing on mine tailings with biochar bioaccumulated a significantly higher Zn concentration in the roots, in respect to those growing on mine tailings. Over exposure time, Zn concentration in the roots of the plants growing on the mine tailing showed a significant increase. However, for the plants growing on biochar, Zn concentrations in roots were similar at the two sampling times (Figure 2, Table S2).

With respect to Zn bioaccumulation in the aerial tissues of *P. laevigata*, the treatments did not show a significant effect. Plants bioaccumulated similar concentrations of Zn in the aerial tissues at two sampling times regardless of the treatment. Zinc concentration in the aerial tissues of plants growing on mine tailing substrate increased over exposure time, while in the plants growing biochar, Zn concentrations did not increase over time. The treatment and exposure time did not significantly affect Zn translocation in the *P. laevigata* individuals. TF values were higher than one in both treatments at two sampling times (Figure 2, Table S2).

### 2.5. P. laevigata Total Heavy Metals Phytoextraction

**Figure 3:**
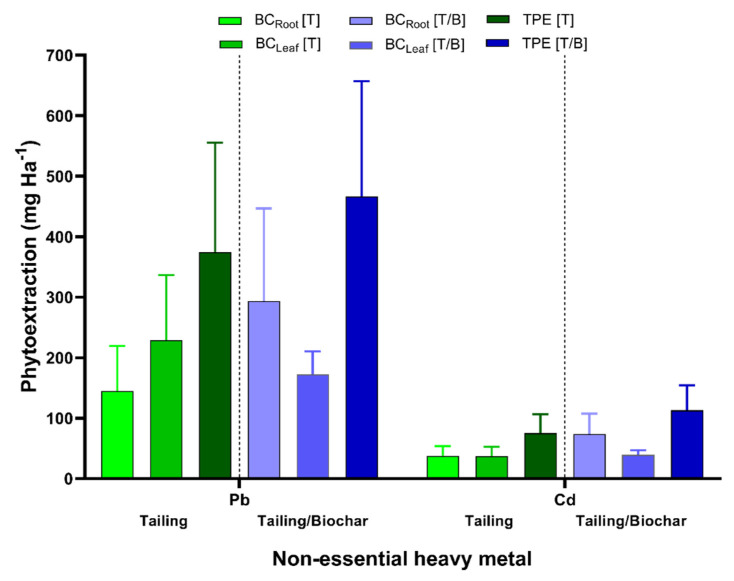

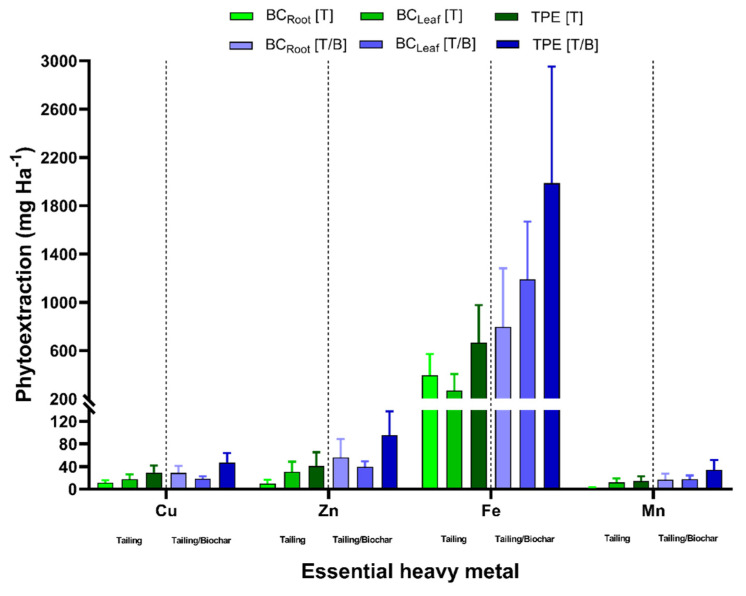
Total heavy metals phytoextraction in *P. laevigata* growing on tailing (T) and tailing/biochar (T/B) in in situ conditions for 180 days. The plant arrangement in the field plot was 2 × 2 m, BC: heavy metal bioconcentration per hectare after 180 days, TPE: total phytoextraction per hectare after 180 days.

After 180 days of exposure, *P. laevigata* plants growing in the mine tailing treatment showed total phytoextraction values for non-essential metals of 75.07 ± 31.5 mg/ha for Cd and 374.40 ± 181.0 mg/ha for Pb. While the total phytoextraction values for essential metals were 14.52 ± 8.3 mg/ha for Mn, 29.25 ± 12.7 mg/ha for Cu, 40.80 ± 24.5 mg/ha for Zn, and 664.22 ± 311.1 mg/ha for Fe. Concerning the plants growing in the mine tailing/biochar mixture treatment, the total phytoextraction values increased for all metals evaluated, being 34.24 ± 17.4 mg/ha for Mn, 46.96 ± 17.1 mg/ha for Cu, 113.19 ± 41.2 mg/ha for Cd, 466.37 ± 190.8 mg/ha for Pb and 1986.48 ± 965.5 mg/ha for Fe. Both treatments observed the highest total phytoextraction for Pb and Fe (Figure 3, Table S3).

## 3. Discussion

### 3.1. Physicochemical Properties of Biochar

According to the physiochemical analysis, the coconut fiber/corn cob biochar has a strong alkaline pH value. In previous reports, the pH values of corn cob biochar showed a highly alkaline profile reporting value of 8.7 [46] and 10.3 [47]. Meanwhile, the biochar produced from coconut fiber at 500 and 700 °C showed pH values of 10.3 and 10.5, respectively, while in the reports of Li et al. [48] the pH of the coconut fiber biochar was of 10.3. These high pH values are attributed to the presence of metals such as K, Ca, and Mg in the biochar composition [46,47]. However, other authors mention that the biochar alkalinity is influenced by the presence of organic functional groups (COOH and OH), carbonates (CaCO_3_ and MgCO_3_), and alkaline salts [32,49].

The coconut fiber/corn cob biochar evaluated in this report showed a high carbon content, while the nitrogen content was low, and had a concentration of 10.5 mg/kg for phosphorous. So, the C/N ratio is high (78.8), similarly to the report by Guarnieri et al. [32], where the C/N ratio in coconut fiber biochar was 72.4. Prakongkep et al. [49] reported the chemical characteristics of the biochar were produced by employing different agro-industrial wastes as raw material, including coconut fiber and corn cobs; overall, the carbon percentages in the biochar composition ranged from 46 to 80%, while the nitrogen percentages ranged from 0.3 to 2.3%. The biochar application as an amendment in soils increases the organic carbon percentage [50], which is important to increase the productivity of crop fields, especially in poor-nutrient and overexploited soils. However, some studies report that the application of biochar with a high C/N ratio to soil reduces the availability of nitrogen forms such as NO^3−^ and NH^4+^ [51], as an adverse effect. Moreover, it has been reported that biochar retains a significant proportion of the phosphorus present in the raw material employed in its production; after application to the soil, the biochar dissolution makes phosphorus available as nutrients for plants [52]. Furthermore, the addition of biochar to soil increases phosphorus availability by up to 45% [51]. It has been documented that biochar addition to soil increases the abundance, speciation, and availability of P in soils, while reducing the P leaching loss, such beneficial effects on P availability can be due to: (1) the P input to the soil from biochar composition, in which soluble and exchangeable P is present; (2) the enhancement of the endogenous P availability in soils by biochar, due to P complexation and by promoting the phosphate-solubilizing microorganism metabolism; and (3) biochar reduces the leaching P losses from the soil through absorption due to its porous structure, large internal surface area, and high water retention capacity, thus increasing the P retaining in soil and favoring plant P uptake. Hence, the use of biochar as an amendment in phytostabilization approaches may be beneficial for plant development, due to its positive effect of increasing the soil P availability and favoring P plant uptake [53].

### 3.2. Size Characters of P. laevigata

*P. laevigata* plants growing on the mine tailing with biochar showed higher values for the basal diameter and the fresh and dry biomass of roots compared to the plants growing on the mine tailing substrate. Similar results show that the addition of biochar in soils contaminated with HM has positive effects on the growth and on biomass development of plants distributed in these sites concerning those plants grown on the substrate without biochar [54,55]. These findings highlight that the addition of biochar on polluted substrates enhances plant grow and biomass development which facilitates plants to cope with HM exposure and therefore use for phytostabilization strategies.

The exposure of *P. laevigata* individuals to mine tailing substrate caused a significant reduction in the FRB and FAB values over time. In contrast, this effect was not documented in plants grown on biochar. The biomass reduction observed in the *P. laevigata* individuals exposed to mine tailings can be attributed to the presence of HM in the substrate. Similar results were achieved by Muro-González et al. [23] and Buendía et al. [42]. The reduction in size characters observed in the mine tailing exposed plants can be related to HM bioaccumulation, since they interfere with important metabolic processes in plants, resulting in growth anomalies [56]. Maldonado-Magaña et al. [57] observed that the absorption of Pb causes a decrease in the growth rate of the roots and affects the branching pattern in *Acacia farnesiana* L. Willd. Additionally, Shanker et al. [58] mentioned that Cr bioaccumulation inhibited root growth in *Caesalpinia pulcherrima* (L.) Sw., *Triticum aestivum* L., and *Vigna radiata* (L.) R. Wilczek. These findings are important because they highlight that even though *P. laevigata* individuals are visibly affected by HM bioaccumulation, these native plants can remain useful for phytoremediation strategies without causing their death. 

On the other side, the reduction in the fresh biomass values of root and aerial tissues of *P. laevigata* plants exposed to mine tailings could also be attributed to an effect of the hydric stress caused by drought season at the time of the second sampling. In the plant growing on the presence of biochar, the biomass values in roots and aerial tissues were not affected by the drought conditions; this could indicate that biochar adding to the mine tailing substrate retains the soil moisture minimizing the hydric stress. Saffari et al. [59] observed that the presence of biochar from corn residues in soils improved water retention in the substrate and increased its availability. Similarly, Dhar et al. [60] suggested that biochar produced from coconut fiber through a pyrolysis process (500–600 °C), has employment potential as an organic soil amendment for water retention.

### 3.3. Bioaccumulation and Translocation of Nonessential Heavy Metals in P. laevigata

Cadmium bioaccumulation in roots and aerial tissues was higher in plants exposed to mine tailing substrate, Cd concentration in both tissues also increased over time, while the plants growing on biochar showed a contrary effect. According to these findings, the addition of biochar to mine tailings reduces the bioaccumulation of Cd in *P. laevigata* tissues. It is important to highlight that even that the tailing/biochar substrate had 12% less Cd than the tailing substrate, the bioaccumulation in roots and leaves was less than the expected proportion without biochar. Hence, this type of biochar contributes to cadmium phytostabilization in soils. Different studies document a reduction in Cd bioaccumulation in plants when biochar is added to the substrate [35,56]. In general, the addition of biochar to the soil increases the overall pH in soil, alkaline pH conditions conduct to the Cd ions precipitation as Cd(OH)_2_, reducing its bioavailability in soils [61]. In the study by Muro-González et al. [23], *P. laevigata* plants exposed ex situ to mine tailings from the Huautla mining district, did not show Cd bioaccumulation in their tissues, thus contrasting to the findings of the present study. Hence, this discrepancy can be attributed to the different experimental conditions used (ex situ vs. in situ). In in situ studies, the tailing samples are homogenized, resulting in more consistent HM concentration across samples, contrasting to in situ studies where the individuals grow directly on the tailing, which can have different HMs, resulting in some plants showing more Cd bioaccumulation than others.

With respect to Pb bioaccumulation, the *P. laevigata* plants exposed to mine tailing substrate showed higher Pb concentrations in both root and aerial tissues with respect to plants growing in the presence of biochar, Pb concentration increased over exposure time, as well as the registered TF which was higher than 1. It is important to highlight that even though the tailing/biochar substrate had 12% less Pb than the tailing substrate, the bioaccumulation in roots and leaves was less than the expected proportion without biochar. Hence, this type of biochar contributes to lead phytostabilization in soils. Muro-González et al. [23], observed that Pb bioaccumulation in *P. laevigata* plants exposed ex situ to mine tailings from Huautla, Morelos, increased according to the exposure time (2, 4, and 6 months). Although Pb is not an essential element for plant nutrition and development, when it is bioavailable in the soil, it is commonly absorbed by roots through the apoplastic pathway or the employment of ion channels and then translocated to aerial tissues of plants [62]. It has been documented that the presence of biochar in HM polluted substrates reduces Pb bioaccumulation in plant tissues (roots and leaves). Li et al. [63], observed a reduction in the bioaccumulation of Pb in rice, when plants were grown on a Pb-enriched soil supplemented with coconut fiber–biochar, the effect was explained by a reduced Pb bioavailability which generated a strong interaction between Pb ions, the biochar surface, and the formation of chemical compounds such as Pb_3_(PO_4_) and Pb carboxylates [64].

### 3.4. Bioaccumulation and Translocation of Essential Heavy Metals in P. laevigata

*P. laevigata* plants established in the mine tailing substrate bioaccumulated higher Cu concentration in the aerial tissues compared to those plants exposed to the mine tailing–biochar substrate. However, Cu concentrations were reduced in the aerial tissues of the plants of both treatments. These findings contrast with those of Muro-González et al. [23], in which an increase in Cu bioaccumulation was observed in the aerial tissues of *P. laevigata* growing on mine tailing substrate from Huautla, Morelos, according to the exposure time, while Cu bioconcentration was reduced in the roots of these plants. Lu et al. [35] reported that the presence of bamboo and rice straw biochar in soils reduces the bioavailability of Cu ions. Eissa [65] observed a lower Cu bioaccumulation in different tissues of *Cucurbita pepo* L. growing on soils supplemented with cow manure biochar compared to Cu bioaccumulation in plants growing on a substrate without biochar. In another study, Zong et al. [66] observed a reduction in the mobility and bioavailability of the Cu fraction in soils as a result of the application of biochar generated from different sources (wheat straw, corn stalk, and rice husk), as a consequence, Cu bioaccumulation was reduced in rice, corn, and radish plants. Biochar presents a great number of C=O functional groups that can interact with Cu ion reducing its bioavailability in soils [66].

In respect to Fe bioaccumulation, plants exposed to mine tailing substrate bioaccumulated higher concentrations in the roots, while the plants growing on biochar bioaccumulated more Fe in the aerial tissues. Muro-González et al. [23] reported higher Fe concentration in the roots of plant exposed to mine tailings from Huautla, Morelos. Similarly, Santoyo-Martínez et al. [27] reported higher Fe concentrations in the roots of the arboreal plant *Vachellia campechiana* (Mill Seigler and Ebinger) exposed to mine tailings from the same site. The lower Fe concentrations observed in the roots of *P. laevigata* plants growing in the presence of biochar could be related to an increase in the pH of the substrate as a result of the addition of this organic amendment of alkaline nature. Moreover, Fe bioavailability decreases in the presence of alkaline substrates [32], while at lower pH values, Fe solubility and bioavailability increases, due to a reduction from Fe III to Fe II [61,67]. Once Fe is absorbed in the roots, it is translocated to aerial tissues, such as leaves, in which Fe demand is higher. On deficiency, Fe translocation is preferential from roots to young leaves [67]. This fact could explain the higher Fe concentration observed in aerial tissues of plants exposed to mine tailing/biochar, in which the Fe bioavailability is lower compared to the mine tailing substrate, thereby increasing the translocation rates.

The healthy growth of plants requires small amounts of Mn, however, an excess of this element may cause toxic effects over plants [68]. During exposure, *P. laevigata* plants growing on both treatments increased their Mn concentrations in roots and aerial tissues. After six months of exposure, the plants growing on biochar showed higher Mn concentrations in the roots, while Mn bioaccumulation in the aerial tissues was not affected by any of the treatments, similar Mn concentrations were observed in the plants growing on both treatments. Similarly, Abbas et al. [69] observed an increase in Mn concentrations in roots, shoots, and grains of wheat plants established in HM polluted soils supplemented with rice straw biochar as organic amendment. Although alkaline pH in soils reduces HM mobility and availability, including Mn, in this study the addition of coconut fiber/corn cob biochar with a pH value around 10 to the mine tailing substrate, favored Mn bioaccumulation in roots of *P. laevigata*. 

Zinc is an essential micronutrient involved in a wide variety of plant physiological processes, although Zn in excess is toxic for plants [70]. In this study, plants exposed to the mine tailing substrate showed a lower Zn concentration in roots and aerial tissues over exposure time, contrary to the plants growing on mine tailing/biochar substrate. In the study by Muro-González et al. [23], a decrease in Zn bioaccumulation was observed in *P. laevigata* roots during exposure time to mine tailings from Huautla, Morelos, as well as the Zn concentration in these plants was higher in the roots than in the leaves, while in the present research, Zn bioaccumulation was higher in the aerial tissues of plants exposed to mine tailing substrate. In this study, the treatment did not show a significant effect over Zn bioaccumulation in *P. laevigata* plants, contrary to what was expected at the beginning of this research. Different researchers mention that the addition of biochar from natural sources such as sugarcane grass, rice husks, and bamboo to soils reduces the overall mobility and bioavailability of Zn [35,54]. However, Fellet et al. [71] did not observe significant differences in Zn bioaccumulation between plants growing on mine tailings, and those growing on the presence of different proportions of biochar from pruning residues, spruce trees, and manure in the substrate.

Concerning the total HM phytoextraction, the corncob/coconut biochar addition to mine tailing substrate causes a positive effect in biomass production in *P. laevigata*, resulting in higher phytoextraction values for all the analyzed HM, Fe and Pb being the metals that showed the higher phytoextraction values after 180 days of exposure in both treatments. In the present study, for total heavy metal phytoextraction determinations, a 2 × 2 m plant arrangement was considered for the field plot, equivalent to 2500 plants per hectare. However, in phytostabilization studies, arrangements with closer distances between plants have been tested, for example, a 0.4 × 0.4 m (60,000 plants·ha^−1^) arrangement was considered for the determination of Cd, Cu, Ni, Pb, and Zn phytoextraction by the plant species *Brassica rapa* L., *Cannabis sativa* L., *Helianthus annuus* L., and *Zea mays* Gaertn, in the presence of soil amendments such as ethylenediamine tetra-acetic acid (EDTA) or ethylenediamine di-succinic acid (EDDS). The evaluated plants can produce 10–20 ton/ha of dry weigh biomass, the highest total phytoextraction was observed for Zn removal with a range of 0.3–1.1 mg·kg^−1^ of soil [72]. In another study, using a 0.2 × 0.2 m (250,000 plant·ha^−1^) arrangement for Cd, Pb, and Zn phytoextraction by the plant species *Dianthus chinensis* L., *Rumex acetosa* L., *Rumex crispus* L., *Sedum alfredii* Hance, *Vetiveria zizanioides* (L.) Nash, and *Viola baoshanensis* W.S.Shu, W.Liu and C.Y.Lan in the presence of EDTA as amendment, the dry weigh biomass production by hectare ranged from 2.5 to 30 ton·ha^−1^, the highest HM phytoextraction values were observed for Zn, 37.2 kg ha^−1^ without soil amendment and around to 50 kg ha^−1^ in presence of EDTA [73]. Felker et al. [74] determined the dry weight biomass production in different *Prosopis* sp. after a year from germination in a 1.5 × 1.5 m arrangement in field plots, the biomass production ranged from 7 to 14.5 ton·ha^−1^. Hence, arrangements with a higher number of plants in field plots can increase the total HM phytoextraction and phytostabilization by *P. laevigata* in phytoremediation field experiments.

Phytostabilization strategies can have a positive effect on stabilizing HM, which are potentially toxic. It reduces contaminants’ bioavailability using plants, often in combination with soil amendments [75,76]. Soil amendments strongly reduce the availability of HM to plant uptake limiting eventual toxicity to plants, allowing revegetation of polluted soils. Establishment of a vegetative cover significantly reduces HM leaching to groundwater and prevents the dispersal of these toxic elements through wind and water erosion from less vegetated sites [76,77], reducing potential risks to ecosystem health.

## 4. Materials and Methods

### 4.1. Study Site

**Figure 4:**
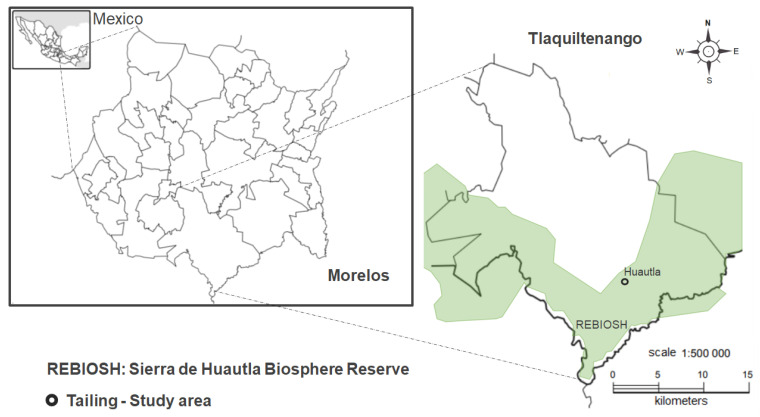
Geographical distribution of the study site at the Sierra de Huautla Biosphere Reserve, Morelos, Mexico.

The present study was carried out in an abandoned mine district located in the community of Huautla, in the state of Morelos, Mexico (8°26′36.37″ N and 99°01′26.71″ W, at an altitude of 974 m). In this area, because of mining activities, around 780 thousand tons of mining wastes rich in HM and metalloids, such as As, Cd, Cr, Cu, Fe Pb, Mn, and Zn, were disposed without any environmental attention. Additionally, this polluted site is in the vicinity of Huautla town, a fact that poses a human and environmental risk for this region (Figure 4).

### 4.2. Plant Species

*Prosopis laevigata* (Humb. et Bonpl. ex Willd) M.C. Johnston (Fabaceae), is an arborous species with natural distribution and dominance in the polluted sites of Huautla, Morelos. This plant is considered as an HM accumulator species [23,41,42,43,44,45]. The *P. laevigata* seeds were collected in the site of Quilamula, Morelos (18°30′52″ N and 98°59′59″ W, at an altitude of 1070 m); this site shares geographical and ecological characteristics with the Huautla site, where mine tailings were deposited [72], but without records of mining activities or HM pollution associated to human activities [11]. For germination, seeds were mechanically scarified to break their physical dormancy, planted in plastic seedbeds with 128 cavities (53 × 27 × 4.4 cm, 19 mL cavity volume), and kept in greenhouse-controlled conditions. After three months, the plants were translated to the experimental plots located on Huautla mine tailings.

### 4.3. Biochar Production and Physicochemical Characterization

In this study, biochar was produced from coconut fiber and corncobs from agro-industrial wastes at 600 °C without oxygen through a pyrolysis process. In the in situ experimental procedures, a 50% mixture of both biochar types was employed. For physicochemical characterization, biochar was considered as soil, pH, organic matter percentage, total carbon, and nitrogen determinations were carried out according to the Mexican standard NOM-021-SEMARNAT-2000. In the present study biochar was produced at 600 °C through a pyrolysis process, using corncobs and coconut fiber as feedstocks, according to the concentrations of total polycyclic aromatic hydrocarbons (PAHs) in similar feedstocks of biochar and to the pyrolysis temperature employed, the concentration of PAHs could range from 0.16 to 1.9 mg·Kg^−1^, concentrations below the maximum permissible concentration (4 mg·Kg^−1^) for premium grade biochar [78].

### 4.4. In Situ Phytostabilization Design

For the in situ phytostabilization strategy, two experimental treatments were evaluated. In the first treatment, 50 plants (5 cm height) were transplanted to 20 cm depth holes in the experimental plots directly on the mine tailing substrate (2 m distance between them). In contrast, in the second treatment, 50 plants (5 cm height) were transplanted to 20 cm depth holes in the experimental plots adding 10% of the biochar mixture, in a completely randomized design, ten plants of each treatment were randomly sampled at three and six months after experimental plots transplantation. Sampling times were established to avoid the roots of the plants exceeding the depth of the mine tailing and biochar mixture, guaranteeing the contact between the roots and the mixture during all experimental evaluations. Metal concentrations in mine tailing substrate were (mg/kg): Fe (80.67), Pb (67.4), Cd (23.78), Cu (7.78), Zn (2.14), and the HM concentration in the substrate mine tailing/biochar were Fe (82.93), Pb (59.31), Cd (20.92), Cu (7.49), and Zn (10.59). In particular, we did not detect the presence of non-essential HM in corncobs/coconut fiber biochar.

### 4.5. Plants size and Biomass Determinations

To evaluate the effect of biochar addition over the establishment and development of plants, seven size and biomass characters were evaluated: aerial part length (AL), root length (LR), basal diameter (BD), fresh aerial part biomass (FAB), fresh root biomass (FRB), dry aerial part biomass (DAB), and dry root biomass (DRB).

### 4.6. Heavy Metal Determination

For HM concentration determinations in *P. laevigata* roots and leaves, 20 plants were randomly selected (10 per treatment/5 plants per exposition time). After sampling, total roots and leaves were separated and washed twice with distilled water to remove substrate and dust remnants. Subsequently, clean tissues were oven-dried (60 °C), the dry tissues were ground, and 250 mg samples of each tissue were acid-digested, adding 10 mL of aqueous HNO_3_ (70% *v*/*v*) in a Microwave Accelerated Reaction System (CEM^®^ MARS-5); an additional sample without tissue was simultaneously processed in the same conditions as a control. Once digested, samples were solubilized in distilled water to 50 mL of final volume, the tissue concentration of six heavy metals was calculated (Cd, Cu, Fe, Mn, Pb, and Zn) through Atomic Absorption Spectrophotometry (GBC-908-AA, Scientific equipment). According to the manufacturer, the equipment detection limits for each metallic element in mg·kg^−1^ were Cd (0.0004), Cu (0.001), Fe (0.005), Mn (0.0015), Pb (0.001), and Zn (0.0005). For concentration determinations, calibration curves for each heavy metal were generated employing known concentration standard solutions for each evaluated metal, and prepared with pure metal ions (ULTRA Scientific, North Kingstown). All standard calibration curves showed correlation coefficients (*R*^2^) between 0.99 and 1.0. In each sample, the heavy metal concentrations were determined by triplicate and reported as the average of such determinations in milligrams/kilogram dry weight (mg/kg DW).

### 4.7. Heavy Metal Translocation Index and Total Phytoextraction

The translocation of HM from roots to leaves was evaluated through the translocation factor (TF) according to Equation (1), TF values higher than 1 indicate heavy metal translocation.

TF = [HM]LT/[HM]RT (1)

where: TF = translocation factor; [HM]LT = heavy metal concentration in leaf tissue [mg·kg^−1^ DW]; [HM]RT = heavy metal concentration in root tissue [mg·kg^−1^ DW].

The total phytoextraction potential per hectare of *P. laevigata* for each heavy metal tested after 180 days of treatment, was calculated according to Equation (2), for TPE determinations, the plant arrangement in field plots was 2 × 2 m, equivalent to 2500 plants per hectare.

TPE = [BA_root_ × BDW_root_] + [BA_leaf_ × BDW_leaf_] (2)

where TPE is the total phytoextraction for each heavy metal [mg·ha^−1^]; BC_root_ is the heavy metal bioconcentration in root tissues [mg·kg^−1^ DW]; BDW_root_ is the dry weight of root biomass per hectare [kg·ha^−1^]; BC_leaf_ is the heavy metal bioconcentration in leaf tissues [mg·kg^−1^ DW]; and BDW_leaf_ is the dry weight of leaf biomass per hectare [kg·ha^−1^].

### 4.8. Statistical Analysis

For statistical analysis, the normality of the experimental data set was evaluated through the Shapiro–Wilk “W” test [79]. Subsequently, two-way analysis of variance (ANOVA) was utilized to evaluate the treatment effect (mine-tailing substrate and 10% biochar addition) and the exposure time (3 and 6 months) over the heavy metal bioaccumulation and the size characters and biomass of the *P. laevigata* individuals. Afterward, a post-hoc Tukey test (*p* < 0.05) was conducted to determine significant differences between pairs of average values among the exposure times for each treatment [79]. The statistical analyses were carried out in the *R* platform [80]. Finally, regression analyses were carried out to test the relationship between the bioaccumulation of each evaluated heavy metal and the root length development over time in both treatments. The software used for statistical analysis were STATISTICA 8.0 [81].

## 5. Conclusions

The *in situ* application of coconut fiber/corn cob biochar to mine tailings favored the establishment of *P. laevigata* plants in the HM polluted site by increasing their biomass and basal diameter. In addition, biochar retained the moisture in the substrate, favoring the development of plant fresh biomass. Concerning the HM bioaccumulation, the biochar addition increased the bioaccumulation of essential metals such as Fe and Mn in *P. laevigata* tissues while reducing the bioaccumulation and translocation of Cu and the non-essential and most toxic metals, Pb and Cd. Therefore, it is suggested that the use of biochar from coconut fiber and corn cob can be an appropriate methodology to facilitate the establishment of plants for phytostabilization of contaminated soils, especially those with non-essential heavy metals such as Pb and Cd. Moreover, the addition of biochar improves HM phytostabilization in soils in comparison with other approaches such as compost which improves phytoextraction efficiency

However, it would be convenient to carry out more in situ studies to prove that *P. laevigata* is applicable for phytoremediation processes (it is capable of accumulating heavy metals) when the roots of the plants exceed the depth of the biochar mixture. Additionally, it would be interesting to analyze the benefits of adding the biochar generated from other plant sources to mine tailings in order to enhance phytoextraction and phytoremediation processes.

## Data Availability

Data recorded in the current study are available in all tables of the manuscript.

## Supplementary Materials

The following supporting information can be downloaded at: https://www.mdpi.com/article/10.3390/plants11243441/s1, Table S1: Mean ± standard deviation and Two way ANOVA results for size and biomass characters for *P. laevigata* growing on tailing and tailing/biochar in in situ conditions.; Table S2: Average ± standard deviation and Two way ANOVA results for heavy metal concentration (mg∙Kg^−1^) in roots and leaves of P. laevigata growing on tailing and tailing/biochar in *in situ* conditions; Table S3: Total heavy metal phytoextraction in *Prosopis laevigata*.
