# Supplementary material for: Assisted Phytostabilization of Mine-Tailings with Prosopis laevigata (Fabaceae) and Biochar
Source: Plants (Basel). 2022 Dec 9;11(24):3441. doi: 10.3390/plants11243441 (PMC9784783; doi:10.3390/plants11243441)
Supplement: Supplementary file 1 [file plants-11-03441-s001.zip › Supplementary Table S1.pdf]

Table S1. Mean ± standard deviation and two ways ANOVA results for size and biomass characters for *P. laevigata* growing on tailing and tailing/biochar in *in-situ* conditions.

Character	Units	Treatment (T)	Exposure time (days)				SDt	ANOVA	
			90		180				$F_{1,15}$
Aerial part length	cm	Tailing	9.03±0.68	a	9.13±3.71	A	ns	Treatment (T)	2.245 n.s.
		Tailing/Biochar	11.80±4.25	a	10.75±1.50	A	ns	Time (t):	0.105 n.s.
								T × t:	0.153 n.s.
Root length	cm	Tailing	18.05±7.92	a	8.25±2.63	A	ns	Treatment (T)	2.932 n.s.
		Tailing/Biochar	17.93±3.05	a	20.50±11.03	A	ns	Time (t):	1.041 n.s.
								T × t:	3.054 n.s.
Basal diameter	mm	Tailing	1.86±0.16	b	1.51±0.37	B	ns	Treatment (T)	15.786 **
		Tailing/Biochar	2.37±0.33	a	2.09±0.18	A	ns	Time (t):	5.256 *
								T × t:	0.080 n.s.
Fresh aerial part biomass	g	Tailing	0.300±0.000	b	0.169±0.086	A	*	Treatment (T)	7.805 *
		Tailing/Biochar	0.650±0.332	a	0.308±0.073	A	ns	Time (t):	7.288 *
								T × t:	1.443 n.s.
Fresh root biomass	g	Tailing	0.475±0.096	a	0.139±0.069	B	*	Treatment (T)	11.625 **
		Tailing/Biochar	0.850±0.311	a	0.406±0.178	A	ns	Time (t):	17.131 **
								T × t:	0.324 n.s.
Dry aerial part biomass	g	Tailing	0.113±0.021	a	0.146±0.074	A	ns	Treatment (T)	5.073 *
		Tailing/Biochar	0.163±0.096	a	0.244±0.048	A	ns	Time (t):	3.013 n.s.
								T × t:	0.526 n.s.
Dry root biomass	g	Tailing	0.184±0.057	a	0.109±0.052	B	ns	Treatment (T)	6.923 *
		Tailing/Biochar	0.221±0.086	a	0.309±0.138	A	ns	Time (t):	0.018 n.s.
								T × t:	3.231 n.s.

Different lowercase letters denote significant differences among treatments after 90 days of the exposition (Tukey $P < 0.05$). Different uppercase letters denote significant differences among treatments after 180 days of the exposition (Tukey $P < 0.05$). SDt = Statistical differences between exposition times. ns = not significant differences, ** = $P < 0.01$, *** = $P < 0.001$.
