# Supplementary material for: Assisted Phytostabilization of Mine-Tailings with Prosopis laevigata (Fabaceae) and Biochar
Source: Plants (Basel). 2022 Dec 9;11(24):3441. doi: 10.3390/plants11243441 (PMC9784783; doi:10.3390/plants11243441)
Supplement: Supplementary file 1 [file plants-11-03441-s001.zip › Supplementary Table S2.pdf]

Table S2. Average ± standard deviation and two-way ANOVA results for heavy metal concentration ($mg \cdot Kg^{-1}$) in roots and leaves of *P. laevigata* growing on tailing and tailing/biochar in *in-situ* conditions.

Treatment	Time (days)	Root		Leaf		TF (min – max)	Root		Leaf		TF (min – max)
**Non-essential metals**											
		**Lead (Pb)**					**Cadmium (Cd)**				
Tailing	90	233.31±32.13	a	259.87±30.91	A	1.14 (0.85-1.44)	67.93±3.90	a	65.45±1.31	A	0.97 (0.85-1.03)
Tailing/Biochar	90	231.22±33.31	a	219.98±8.71	A	0.97 (0.75-1.31)	64.84±2.41	a	62.82±1.08	B	0.97 (0.90-1.02)
Tailing	180	375.63±91.02	b	467.76±55.65	B	1.28 (0.94-1.59)	107.62±7.35	b	80.50±1.61	C	0.75 (0.66-0.82)
Tailing/Biochar	180	264.34±78.85	a	250.54±12.74	A	0.96 (0.78-1.13)	71.13±11.58	a	58.42±1.00	D	0.84 (0.64-0.96)
ANOVA: $F_{1,44}$											
Treatment (T)		25.607 ***		184.876 ***			89.861 ***		1133.346 ***		
Time (t)		61.305 ***		159.025 ***			121.253 ***		210.657 ***		
T × t:		23.756 ***		87.952 ***			63.977 ***		701.824 ***		
**Essential metals**											
		**Cooper (Cu)**					**Zinc (Zn)**				
Tailing	90	41.77±1.24	a	49.40±4.68	A	1.19 (0.95-1.41)	58.93±11.84	a	73.56±23.64	A	1.25 (0.75-1.76)
Tailing/Biochar	90	23.20±22.24	b	0.00±0.00	B	0.50 (0.00-1.00)	53.80±21.60	a	60.52±5.09	AB	1.29 (0.82-1.87)
Tailing	180	33.00±0.98	ab	37.05±3.51	C	1.13 (0.91-1.34)	21.57±13.84	b	54.77±20.65	B	3.28 (1.64-6.16)
Tailing/Biochar	180	28.26±3.53	b	25.94±2.46	D	0.93 (0.79-1.23)	46.36±21.43	a	56.28±4.73	AB	1.52 (0.84-2.78)
ANOVA: $F_{1,44}$											
Treatment (T)		10.819 **		1092.823 ***			3.726 n.s.		1.543 n.s.		
Time (t)		0.275 n.s.		55.070 ***			19.343 ***		6.158 ***		
T × t		3.808 n.s.		437.398 ***			8.624 ***		2.461 n.s.		
		**Iron (Fe)**					**Manganese (Mn)**				
Tailing	90	1230.24±123.66	a	559.07±244.52	A	0.50 (0.01-1.00)	0.00±0.00	a	0.00±0.00	A	1.00 (1.00-1.00)
Tailing/Biochar	90	355.66±174.38	b	1480.60±460.21	B	1.19 (0.95-1.41)	1.80±3.34	a	0.00±0.00	A	0.75 (0.00-1.00)
Tailing	180	1094.43±124.70	a	530.18±98.91	A	0.93 (0.79-1.23)	7.27±0.84	b	20.68±8.86	B	2.82 (1.57-5.06)
Tailing/Biochar	180	646.80±333.42	c	1508.35±488.65	B	1.13 (0.91-1.34)	13.48±7.35	c	22.15±6.99	B	2.58 (0.65-6.36)
ANOVA: $F_{1,44}$											
Treatment (T)		121.674 ***		83.260 ***			11.245 ***		0.203 n.s.		
Time (t)		1.679 n.s.		0.000 n.s.			62.867 ***		172.987 ***		
T × t		12.687 ***		0.074 n.s.			3.413 n.s.		0.655 n.s.		

Different lowercase letters denote significant differences among treatments in heavy metal root concentration (Tukey $P < 0.05$). Different uppercase letters denote significant differences among treatments in heavy metal leaves concentration (Tukey $P < 0.05$). TF = translocation factor, n.s. = not significant differences, ** = $P < 0.01$, *** = $P < 0.001$.
