# Supplementary material for: Assisted Phytostabilization of Mine-Tailings with Prosopis laevigata (Fabaceae) and Biochar
Source: Plants (Basel). 2022 Dec 9;11(24):3441. doi: 10.3390/plants11243441 (PMC9784783; doi:10.3390/plants11243441)
Supplement: Supplementary file 1 [file plants-11-03441-s001.zip › Supplementary Table S3.pdf]

Table S3. Total heavy metal phytoextraction in *Prosopis laevigata*

	Treatment	BC_{Root} (mg/Ha)	BC_{Leaf} (mg/Ha)	TPE (mg/Ha)
**Non-essential metals**				
**Lead (Pb)**	Tailing	145.09±74.0	229.30±107.0	374.40±180.96
	Tailing/Biochar	293.86±152.9	172.51±37.8	466.37±190.76
**Cadmium (Cd)**	Tailing	37.80±16.0	37.27±15.5	75.07±31.48
	Tailing/Biochar	73.69±33.6	39.51±7.6	113.19±41.19
**Essential metals**				
**Copper (Cu)**	Tailing	11.33±4.6	17.92±8.1	29.25±12.7
	Tailing/Biochar	28.68±12.5	18.28±4.6	46.96±17.11
**Zinc (Zn)**	Tailing	10.07±6.7	30.74±17.8	40.80±24.46
	Tailing/Biochar	55.78±32.8	39.43±9.6	95.22±42.47
**Iron (Fe)**	Tailing	394.47±176.5	269.76±134.6	664.22±311.11
	Tailing/Biochar	797.52±485.3	1188.95+480.3	1986.48±965.55
**Manganese (Mn)**	Tailing	2.62±1.2	11.90±7.1	14.52±8.30
	Tailing/Biochar	16.85±10.4	17.39±6.9	34.24±17.37

The plant arrangement in the field plot was 2 x 2 m
BC: Heavy metal bioconcentration per hectare after 180 days
TPE: Total phytoextraction per hectare after 180 days
